# Rare-earth fluorescence thermometry of laser-induced plasmon heating in silver nanoparticles arrays

**DOI:** 10.1038/s41598-018-32179-7

**Published:** 2018-09-14

**Authors:** Tiziana Cesca, Giovanni Perotto, Giovanni Pellegrini, Niccolò Michieli, Boris Kalinic, Giovanni Mattei

**Affiliations:** 1 University of Padova, Department of Physics and Astronomy, NanoStructures Group, via Marzolo 8, I-35131 Padova, Italy; 2 Istituto Italiano di Tecnologia, Smart Materials, Via Morego 30, I-16163 Genova, Italy; 3 Dipartimento di Fisica, Politecnico di Milano, Piazza Leonardo da Vinci 32, 20133 Milano, Italy

## Abstract

The laser-induced plasmon heating of an ordered array of silver nanoparticles, under continuous illumination with an Ar laser, was probed by rare-earth fluorescence thermometry. The rise in temperature in the samples was monitored by measuring the temperature-sensitive photoluminescent emission of a europium complex (EuTTA) embedded in PMMA thin-films, deposited onto the nanoparticles array. A maximum temperature increase of 19 °C was determined upon resonant illumination with the surface plasmon resonance of the nanoarray at the highest pump Ar laser power (173 mW). The experimental results were supported by finite elements method electrodynamic simulations, which provided also information on the temporal dynamics of the heating process. This method proved to be a facile and accurate approach to probe the actual temperature increase due to photo-induced plasmon heating in plasmonic nanosystems.

## Introduction

Nowadays plasmonic nanostructures find application in many different fields in nanophotonics, as light harvesting and manipulation^1–3^, fluorescence enhancement^4–7^, nonlinear optics^8–13^, biosensing^14–20^, surface-enhanced Raman scattering (SERS)^21–23^ and medical therapies^24–26^. For most of these applications, photo-induced heating is a key issue. On one hand, indeed, the use plasmonic nanostructures as nano-sources of heat and the engineering of the formation of photo-induced thermal hot-spots is at the basis of the emerging field of thermo-plasmonics^27^, with a variety of applications as optically-assisted drug delivery^28,29^, photo-thermal cancer therapy^24–26,30,31^, heat-assisted nanochemistry^32–34^, and recently the development of plasmonic nano-ovens^35^ or adaptive lenses^36^. On the other hand, unwanted photo-heating may represent a severe drawback in many cases, limiting device performances or causing strong sample modifications^37,38^. For instance when biological molecules are in close proximity to plasmonic nanostructures (e.g., in biosensors or in SERS spectroscopy) local spikes in temperature can cause alteration of their structure and thus their functionality. Moreover, the temperature increase upon external illumination and its spatial distribution in plasmonic nanoarrays were demonstrated to be dependent on the shape, composition and geometrical distribution of the nanostructures, and the conditions of illumination (cw or pulsed)^39,40^. In this context, the estimation of the actual temperature in plasmonic nanosystems is of paramount importance.

Different approaches have been developed during these years to probe the local temperature at the micro- and nano-scale^41^, which include scanning thermal microscopy (SThM)^42^, thermoreflectance microscopy^43^ and temperature-dependent Raman spectroscopy^44^. SThM provides the highest spatial resolution, but its major drawbacks are the slow readout rate and the need of contact between the scanning probes and the samples, which may create a thermal bridge and compromise the accuracy in the temperature measurement. The latter two are non-invasive and non-contact techniques, but are unsuitable for metallic surfaces (Raman) or require lengthy calibration procedures (reflectance). Recently two fluorescence-based techniques demonstrated to be very effective to probe the local temperature in the vicinity of metallic nanostructures: (i) fluorescence polarization anisotropy (FPA)^45^, which exploits the decrease of polarization anisotropy of the emitted fluorescence of specific fluorescent molecules incorporated in a medium of interest at increasing temperatures, and (ii) fluorescence thermometry in which the temperature-dependent emission intensity of a fluorophore, in close-contact with the sample under investigation, is detected^46,47^. In the present work fluorescence thermometry has been employed to probe the local temperature of an array of silver nanoparticles illuminated by a cw laser source, using as a probe a thin film of polymethylmethacrylate (PMMA) doped with the europium(III) thenoyltrifluoroacetonate complex (EuTTA)^48,49^. The high sensitivity of the rare-earth photoluminescence to its environment temperature^50^, its stable and intense emission in the visible range (at ${\lambda }_{E{u}^{3+}}=613$ nm)^51,52^, combined with the ease to couple the thermometric probe with the samples (EuTTA-doped PMMA thin-films can be deposited by spin-coating on the substrates and can be easily removed once the thermal analysis is completed by rinsing the samples in acetone, which will dissolve the film in few minutes without affecting the plasmonic nanostructures) make this method extremely effective for the measurement of the actual temperature increase due to photo-induced heating in plasmonic nanosystems.

## Experimental Section

### Samples preparation

Ordered arrays of silver nanoparticles were synthesized via nanosphere lithography (NSL)^11,53,54^. As the first step, a colloidal monolayer of polystyrene (PS) nanospheres was self-assembled on a sodalime-glass (SLG) substrate, according to the method proposed by Giersig *et al*.^55^. The colloidal monolayers were prepared using commercial PS nanospheres with diameter of 330 nm, purchased from Microparticles GmbH (Germany) as a 10% vol. water dispersion. The sodalime-glass substrates were cleaned with acetone in a ultrasonic bath for 2 minutes and then immersed in a “piranha” solution (3:1 mixture of concentrated sulfuric acid and 30% hydrogen peroxide) for half an hour, rinsed with deionized water and dried in a stream of nitrogen.

Subsequently, silver was deposited over the monolayer of PS nanospheres by magnetron sputtering in orthogonal geometry. The samples were then immersed in toluene (purchased from Sigma Aldrich) for 2 minutes and sonicated for 1 minute to dissolve the PS nanospheres and remove them from the substrate. After this step, an ordered array of silver triangular nanoprisms remains on the glass substrates^10,12,22^. To finely tune the surface plasmon resonance properties of the nanoarray, and to maximize the absorption of the pump laser used to heat the plasmonic nanostructures in the fluorescence thermometry experiment, the samples were further annealed in air for 30 min at 350 °C. This treatment modifies the shape of the Ag nanostructures, which change from triangular nanoprisms to spheroidal nanoparticles, preserving their ordered arrangement.

A film of polymethylmethacrylate (PMMA) doped with the europium(III) thenoyltrifluoroacetonate complex (EuTTA, Acros Organics, 95%) was then deposited onto the Ag nanoparticles array (NPA). To obtain this film, a 10% PMMA (MW = 250.000) solution was prepared leaving PMMA in toluene in a oven at 70 °C overnight. In the same way a 50 mM solution of EuTTA in toluene was prepared (since at such a high concentration EuTTA is not stable at room temperature, the solution was used while warm). The PMMA and EuTTA solutions were then mixed together to obtain a concentration of 25 mM of EuTTA and 0.5% wt of PMMA in toluene. The prepared solution was immediately used for coating the silver nanostructures, using a G3P spin coater (Specialty Coating Systems) at 4000 rpm for 60 s. Two reference samples were also produced by depositing the EuTTA-doped PMMA film on a bare SLG substrate and on a continuous silver film deposited by magnetron sputtering on a sodalime-glass (Ag film thickness: 127 ± 5 nm). The parameters of the different synthesized samples are summarized in Table 1.

**Table 1:** Synthesis parameters of the samples investigated in the present work.

Sample label	Ag layer	EuTTA-PMMA
Eu-SLG	none	20 ± 3 nm
Eu-Ag_film	continuous film (*t* = 127 ± 5 nm)	22 ± 3 nm
Eu-Ag_NPA	nanoparticles array (*h* = 22 ± 3 nm)	20 ± 3 nm

The thickness of the PMMA layer was measured via ellipsometry, while the thickness of the Ag film and the nanoparticles’ height were obtained by AFM measurements.

### Structural and optical characterizations

Scanning electron microscopy (SEM) measurements of the synthesized samples were performed using a Zeiss Sigma HD FE-SEM, operated at 5 kV and with the signal collected by the InLens secondary electrons detector. Atomic force microscopy (AFM) measurements were done using a NT-MDT Solver-PRO AFM microscope operated in non-contact mode.

Optical extinction spectra of the samples were recorded using a JASCO V670 spectrophotometer in the 300–2100 nm range. The spectrophotometer was also equipped with an integrating sphere detector to measure the corresponding scattering spectra. A J. Woolham V-VASE spectroscopic ellipsometer was used to measure the dielectric functions of the EuTTA-doped PMMA layers, the SLG and the Ag film deposited by magnetron sputtering with the same conditions used for the samples.

### FEM simulations

Optical and thermal simulations were carried out using the Finite Elements Method (FEM). The commercial sofware COMSOL Multiphysics was used^18,22^. In the simulations, the honeycomb lattice of the samples in the $\hat{x}y$ plane was modeled by applying periodic boundary conditions (PBC) to a rhombic unit cell. The unit cell contains two plasmonic nanoparticles, which were modeled as oblated ellipsoids in order to reproduce the experimental shape. Consistently with the experimental values measured by SEM and AFM (described below), we setup a series of FEM models varying the size of the axes, to reproduce the dispersion measured on the samples. The results from these models where averaged by assigning to each model the weight given by the distribution. The most probable configuration ellipsoids semi-axes resulted: *a* = *b* = 27 nm in the $\hat{x}y$ plane and *c* = *h*/2 = 11 nm in the $\hat{z}$ direction (*h* is the nanoparticles’ height). Some parts of the samples showed defects and film-like regions. Models for these regions were also solved, and their results contributed to the average spectra with a weight proportional to fraction of sample surface covered by defects and film-like regions.

The simulations were carried out by solving the Helmholtz equation for the unit cell, using periodic boundary conditions for the boundaries orthogonal to the horizontal ($\hat{x}y$) plane. To prevent scattered waves to be reflected back in the solution domain, the scattering boundary conditions were applied for the upper and lower boundaries^56^. The PMMA was modeled as a uniform film that embeds the nanoparticles. Its height was set to 20 nm. The optical properties of silver, SLG and PMMA were described by their dielectric functions, which were measured by spectroscopic ellipsometry. The absorption cross-section was computed as the ratio between the total dissipated power (due to Joule heating) and the incident power flux *P*_0_: ${\sigma }_{abs}=\frac{{\int }_{V}{\bf{E}}\cdot {\bf{j}}\,dV}{{P}_{0}}$, where the integration is carried over the volume of the unit cell.

Coupled to the electrodynamic simulations, a thermal analysis was performed to simulate the heating of the nanostructures. To do this, at each element of the mesh in the nanoparticles the dissipation due to Joule effect was used as a local source of heating for the thermal analysis. Given this source, the heat transfer equations were solved. For the substrate, heat conduction through the SLG substrate was considered, by introducing, as boundary condition, a surface kept at the constant temperature of the surrounding environment and placed at a distance of 1 mm (i.e., the thickness of the SLG substrate) from the sample’s surface. For the physical domain above the sample filled by air, fluid heat transport equations (including conduction and convection) were solved obtaining a contribution of about 20% to the overall dissipation. For the same domain, we also computed the irradiation contribution using the Stefan-Boltzmann law, but in this case, due to the small difference in temperature between the surface and the ambient, its effect resulted negligible. For the thermal simulations, the periodic boundary conditions are the same as the optical ones. The thermal properties of the materials (in particular thermal conductivity, *k*, and specific heat capacity, *c*_*p*_) were taken from available values in COMSOL and were: *k* = 430 Wm^−1^ K^−1^, *c*_*p*_ = 235 J kg^−1^ K^−1^ for Ag; *k* = 0.19 Wm^−1^ K^−1^, *c*_*p*_ = 1420 J kg^−1^ K^−1^ for PMMA; *k* = 1.05 Wm^−1^ K^−1^, *c*_*p*_ = 720 J kg^−1^ K^−1^ for SiO_2_. The simulations were then carried out to get the temperature of the system both in the stationary and transient regime.

### Photoluminescence measurements

A pulsed *N*_2_ laser (emission wavelength ${\lambda }_{{N}_{2}}=337$ nm, pulse duration 500 ps, energy 100 *μ*J) was employed to excite the Eu^3+^ luminescence of the EuTTA-doped PMMA layers close to the maximum of its excitation peak. The laser was operated in burst mode, with 128 pulses per burst at a frequency of 2 Hz. A cw multi-line Ar laser was employed as the pump source for heating the samples. The three most intense lines of the Ar laser (concentrating about 70% of the total emitted power) are at 514.5 nm and 488 nm and 476.5 nm; no line selection was done for this experiment in order to maximize the incident power, exploiting the spectral width of the LSPR resonance. The total power of the laser could be continuously varied up to the maximum value of 173 mW. The overall emitted power was monitored by an on-board power meter whose accuracy was also independently verified. The Eu^3+^ luminescent emission at ${\lambda }_{E{u}^{3+}}=613$ nm was collected with a single grating monochromator coupled with a photomultiplier tube (Hamamatsu R928). Three long-pass filters were placed at the entrance of the monochromator to prevent stray light from the pump laser to reach the detector. A Tektronix TDS 7104 digital oscilloscope (bandwidth 1 GHz) was used to record the PL intensity decay as a function of time.

Organic Eu complexes are known to lose their fluorescence properties upon UV irradiation in an oxygen-containing atmosphere, because of the presence of singlet oxygen which may cause the breaking of the complex. For this reason, in order to minimize bleaching, the samples were kept in vacuum conditions, at a pressure lower than 0.5 mbar. Moreover, by studying the bleaching dynamics a double-exponential decay of the Eu^3+^ luminescent intensity was observed, with two characteristic times (given in terms of the number of pulses of the *N*_2_ laser): *τ*_*b*,1_ = 1600 pulses and *τ*_*b*,2_ = 50000 pulses. For this reason, before taking PL measurements the samples were always let under exposure of the *N*_2_ laser beam for 20 minutes with a repetition rate of 20 Hz (corresponding to a total number of pulses *n* = 24000 >> *τ*_*b*,1_). Moreover, for each sample all the measurements were taken in a very short time compared to *τ*_*b*,2_ and a linear correction was applied to the data to compensate for a possible residual bleaching during the measurements.

## Results and Discussion

**Figure 1:**
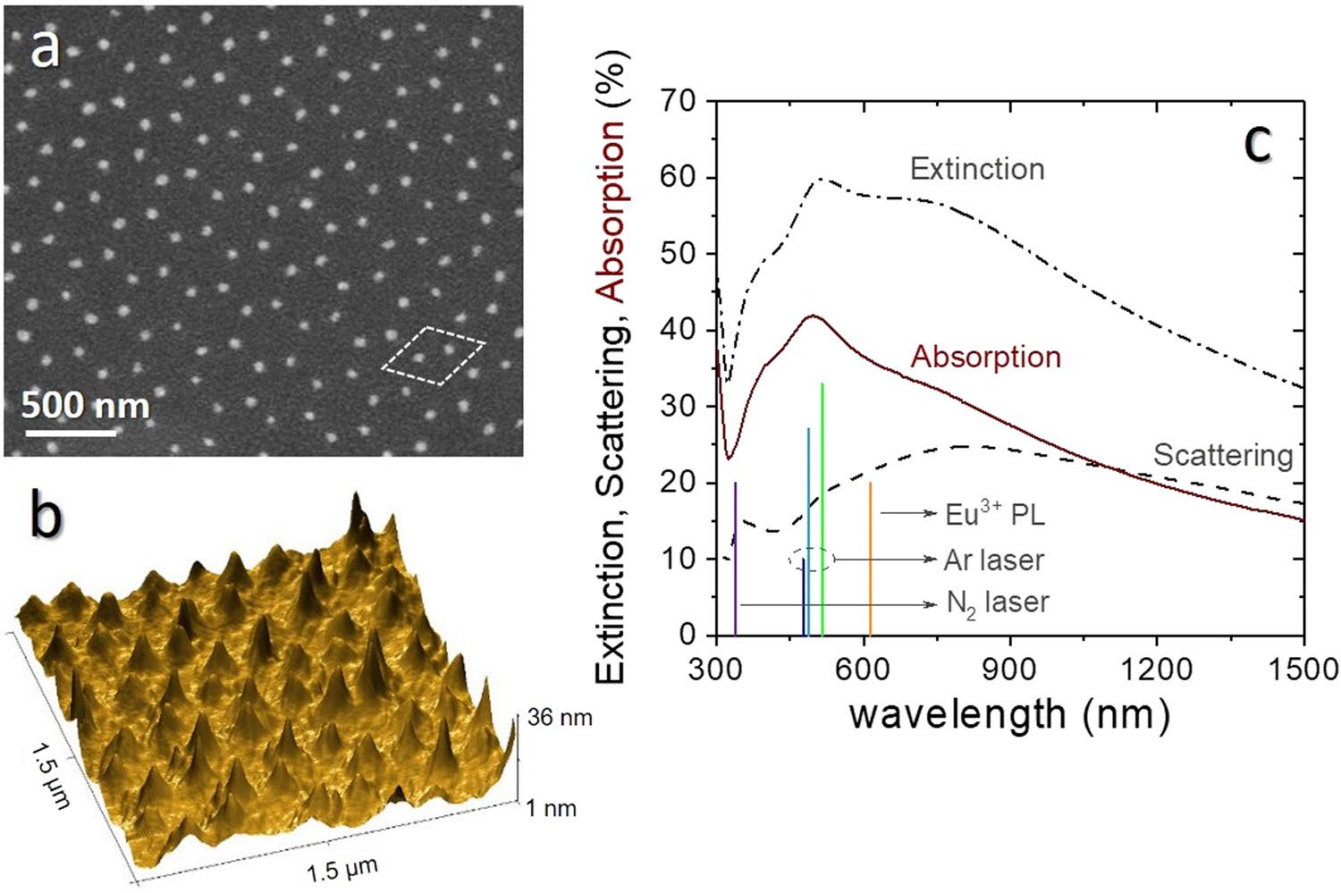
(**a**) SEM image in plane view and (**b**) 3D AFM image of the synthesized Ag nanoparticles array. (**c**) Measured extinction, scattering, and absorption spectra of the Eu-Ag_NPA sample in which the Ag NPA is embedded in the EuTTA-doped PMMA film; the blue and green vertical lines indicate the three most intense lines of the pump Ar laser (476.5 nm, 488 nm and 514.5 nm), while the orange line indicates the EuTTA emission wavelength (613 nm) and the purple line represents the wavelength of the probe N_2_ laser (337 nm).

In Fig. 1a the SEM image in plane view of the synthesized samples is reported; the corresponding 3D AFM image is shown in Fig. 1b. The images show the formation of an ordered array of spheroidal nanoparticles arranged in a honeycomb lattice. From the analysis of these measurements, we determined the geometrical parameters of the nanoparticles: the in-plane radius *R* = 27 ± 3 nm and the height *h* = 22 ± 4 nm. The optical properties of the synthesized samples are reported in Fig. 1c. The extinction and scattering spectra were measured separately using a spectrophotometer equipped with an integrating sphere detector, while the absorption spectrum (red line in Fig. 1c) was obtained by subtracting the scattering contribution from the extinction spectrum. The graph shows that the absorption peak of the sample matches very well the spectral region in which the most intense lines (488 nm and 514.5 nm) of the Ar laser, used to pump the samples in the fluorescence thermometry experiment, are located. In particular, in the wavelength range 450–550 nm, more than 40% of absorption is obtained in the sample, thus giving rise to the corresponding heating of the silver nanoparticles.

**Figure 2:**
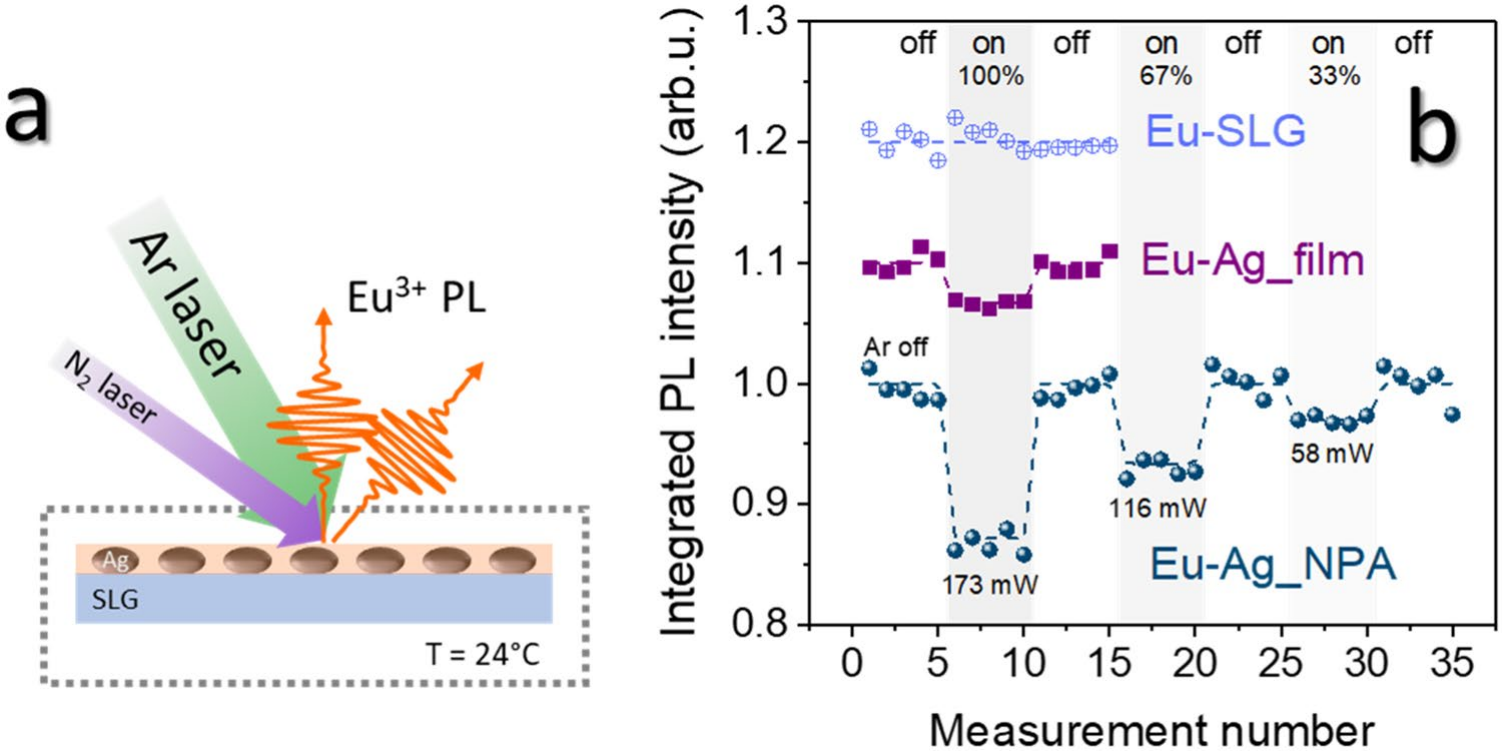
(**a**) Schematics of the experimental configuration: the cw Ar laser was used to heat the plasmonic nanoparticles by absorption at their LSPR, while the N_2_ pulsed laser was used as the probe, to excite the Eu^3+^ luminescence; during the measurements, the samples were placed in a cryostat, under a vacuum (P < 0.5 mbar), and the sample holder was kept at a constant temperature of 24 °C. The diameter of the illuminated spot was 1 mm. (**b**) Eu^3+^ luminescence intensity upon illumination with the pump Ar laser at different powers, for the three samples investigated. A reversible, power-dependent drop of the Eu^3+^ luminescent intensity occurs when the EuTTA-doped PMMA layer is deposited on top of the silver nanoparticles array (sample Eu-Ag_NPA); a much smaller effect (detectable at the highest pump power only) appears for EuTTA on the homogeneous silver film (sample Eu-Ag_film), while no effect was observed from the reference sample in which EuTTA-doped PMMA was deposited on a soda-lime glass (sample Eu-SLG).

**Figure 3:**
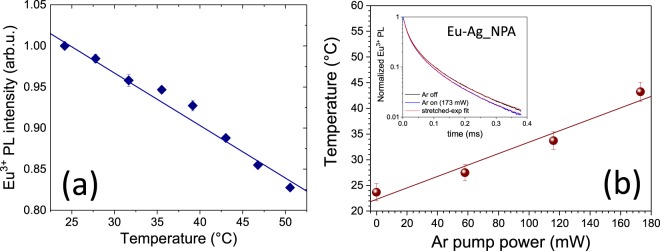
(**a**) Calibration curve of Eu^3+^ PL emission intensity *versus* temperature. (**b**) Temperature as function of the power of the pump Ar laser of sample Eu-Ag_NPA. Inset: PL temporal decay of the sample with the pump Ar laser off (black curve) and on (173 mW, blue curve); the light-red lines are the best stretched-exponential fits. The diameter of the illuminated spot was 1 mm.

In order to investigate the heat-transfer process and to measure the rise of temperature of the plasmonic nanostructures upon laser illumination, we performed a pump-and-probe experiment using the multi-line cw Ar laser as the pump source to excite the LSPR of the plasmonic nanoparticles arrays, thus inducing the heating of the nanoparticles. The N_2_ pulsed laser was used as the probe, to excite the Eu^3+^ luminescent emission of the EuTTA-doped PMMA layers. EuTTA, similarly to other *β*-diketonate complexes, exhibits a narrow emission band that can be excited upon UV excitation via a Dexter intra-molecular energy-transfer from the lowest triplet excited state of the ligands to the quasi-resonant energy state of the rare-earth ions (antenna effect). This step is crucial in determining the quantum yield and thus the temperature dependence of the Eu^3+^ luminescence intensity^50^. A sketch of the measurement configuration is reported in Fig. 2a. Particular care was taken to align the pump (Ar) and probe (N_2_) laser beams on the same spot on the sample (the diameter of the illuminated spot was 1 mm), to make sure that the measured Eu^3+^ PL signal was coming from the heated zone. During the measurements, the samples were placed in a cryostat with quartz windows, at a pressure lower than 0.5 mbar, and an external heater was used to maintain the sample holder at the constant temperature of 24 °C. In Fig. 2b we reported the temporally integrated Eu^3+^ photoluminescence (PL) intensity (at ${\lambda }_{E{u}^{3+}}=613$ nm) of the three samples–Eu-Ag_NPA, Eu-Ag_film and the reference sample Eu-SLG–measured switching on and off the pump Ar laser. To excite the Eu^3+^ luminescence, the *N*_2_ laser (probe) was operated in the burst mode, with 128 pulses per burst; the Eu^3+^ PL signal was normalized to the energy of the *N*_2_ laser which was simultaneously measured with an external photodiode. In the “laser on” configuration, the measurements were taken for three different values of the Ar laser power, corresponding to full power (173 mW), 2/3 of the maximum (116 mW) and 1/3 of the maximum (58 mW). Considering that the diameter of the illuminated spot on the sample is 1 mm, these values corresponds to power density values of 22.0 W/cm^2^, 14.7 W/cm^2^ and 7.3 W/cm^2^, respectively. 5 measurements of the Eu^3+^ PL signal were recorded for each configuration. To let the samples thermalize, the pump laser was shined on them for 5 minutes before performing the “laser on” measurements. Then the Ar laser was shut down and the samples were let cool down for 20 minutes before taking the “laser off” measurements. The results in Fig. 2b show that if the EuTTA-doped PMMA layer is deposited over the Ag nanoparticles array (sample Eu-Ag_NPA), the Eu^3+^ PL signal exhibits a drastic decrease in intensity when the sample is illuminated by the pump laser. The effect is completely reversible and proportional to the pump power. A similar behavior is observed also with the sample Eu-Ag_film, in which the EuTTA-doped PMMA layer was deposited over a homogeneous silver film, but the PL intensity drop is much smaller compared to the nanostructured sample, and can be detected only using the pump laser at full power; no effect is visible at lower pump powers. Finally, regarding the reference sample Eu-SLG, no reduction of the Eu^3+^ PL intensity is observed when the pump laser is shined on the sample, even for the highest pump power. Such a different behavior among the samples is consistent with the different absorption properties of the three samples in the spectral region of the pump Ar laser. Indeed, in the spectral range 450–550 nm, we estimated an average absorption higher than 40% for the Eu-Ag_NPA sample, while it was about 7% for the Eu-Ag_film sample and less than 3% for the reference sample Eu-SLG. Moreover, the results reported in Fig. 2b clearly show that the Eu^3+^ luminescent emission intensity is correlated with the Ar power and thus can be used to determine the samples’ temperature. A comment has to be made at this point. The PL emission lifetime too was demonstrated to be temperature-dependent and thus could be used to determine the samples’ temperature as well^50^. Nonetheless, even if its measurement can be less affected by stray light or residual bleaching, the PL lifetime sensitivity to temperature changes resulted to be slightly lower than for the PL intensity. To highlight this, the following figures-of-merit can be introduced: ${S}_{I}=\frac{{\rm{\Delta }}I}{{I}_{{\rm{off}}}{\rm{\Delta }}T}$ and ${S}_{\tau }=\frac{{\rm{\Delta }}\tau }{{\tau }_{{\rm{off}}}{\rm{\Delta }}T}$, where Δ*I* = *I*_on_ − *I*_off_ and Δ*τ* = *τ*_on_ − *τ*_off_ are, respectively, the differences in intensity and lifetime of the Eu^3+^ PL emission when the pump Ar laser is turned on and off. *S*_*I*_ and *S*_*τ*_ represent the sensitivity to temperature changes induced by the laser-heating that can be obtained in the two ways, and in the present case we get: *S*_*I*_ = −7 × 10^−3^ °C^−1^ and *S*_*τ*_ = −5 × 10^−3^ °C^−1^. Moreover, it is important to note that the accurate estimation of the lifetime could get complicated if the luminescence temporal decay differs from a single exponential decay, as it is for the samples investigated in the present work. As an example, we reported as inset in Fig. 3(b) the temporal decay of the Eu^3+^ PL intensity of the Eu-Ag_NPA sample detected with the Ar pump laser off and on (at 173 mW). The light-red lines are the best fits to the experimental data obtained with the stretched-exponential decay function: $I(t)={I}_{0}{e}^{-{(t/\tau )}^{\beta }}$; in both cases the stretching parameter results *β* = 0.4 (*β* = 1 represents a single-exponential decay). An effective relaxation lifetime can be defined as ${\tau }_{{\rm{eff}}}=\frac{\tau }{\beta }{\rm{\Gamma }}(\frac{1}{\beta })$ (Γ is the Euler gamma function)^57^, and we get: ${\tau }_{{\rm{eff}}}^{{\rm{off}}}=0.022$ ms (pump Ar laser off) and ${\tau }_{{\rm{eff}}}^{{\rm{on}}}=0.020$ ms (pump Ar laser on at 173 mW). For all these reasons, in the present work we preferred to use the Eu^3+^ PL intensity as the thermometric parameter to estimate the rise in temperature of the samples upon laser irradiation.

In order to determine the actual temperature of the samples from the measurement of the Eu^3+^ luminescence intensity, we calibrated the set-up by changing the sample temperature with an external heating system and recording the corresponding Eu^3+^ PL intensity variations. In Fig. 3(a) we reported the (linear) calibration curve obtained on the sample with Ag NPs (Eu-Ag_NPA) after normalization of the intensity to its value at room temperature. Such a normalization should compensate for the variations in the photoluminescence intensity (due for instance to PL quenching, enhancement or to different sample reflectivity), which could arise in samples with different geometry/composition as those considered in the present case. We verified experimentally that the slope of the calibration curve, (6.4 ± 0.3) × 10^−3^ °C^−1^, is the same, within experimental uncertainties, in the three investigated samples. With this calibration curve we were able to determine the temperature reached by the three samples as a function of the pump laser power. As an example, in the main panel in Fig. 3(b) we plotted the temperature, as a function of the Ar laser power, of the Eu-Ag_NPA sample. The error bars (±1.5 °C) can be considered as an estimate of the temperature resolution of the proposed method, in the range of temperatures explored. The graph shows that the silver nanoparticles array experience a linear increase of the temperature, starting from room temperature, 24 ± 1 °C, up to 43 ± 2 °C when illuminated by the Ar laser at full power (173 mW). In the same way, we determined a maximum temperature of 27 ± 2 °C for the Eu-Ag_film sample, while no increase in temperature was measured for the Eu-SLG reference sample.

**Figure 4:**
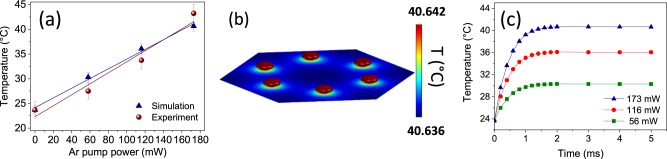
(**a**) Comparison between measured (red dots) and simulated (blue triangles) temperature, as a function of the pump power, of the Ag NPA. (**b**) Simulated map of equilibrium temperature in the nanoarray estimated at the maximum pump power (173 mW); the color bar on the right side shows that the differences in temperature throughout the NPA and between the silver nanoparticles and the surrounding EuTTA-doped PMMA film are almost negligible. (**c**) Temporal evolution of the Eu-Ag_NPA sample temperature for different Ar pump powers obtained by FEM simulations.

As mentioned above, the rise in temperature experienced by the Ag NPA can be related to the strong absorption of the Ar laser beam at the LSPR of the silver nanoparticles array (Fig. 1c). This is confirmed by the results of the thermal analysis performed by electrodynamic simulations. In Fig. 4a we reported the comparison between the measured and FEM simulated temperature of the silver nanoparticle array for the different pump laser powers. An optimal agreement between the simulations and the experimental data is obtained. Moreover, the results are consistent with the model proposed by Baffou and co-workers for photo-induced heating of plasmonic nanoparticle arrays upon continuous illumination^40^. According to Baffou’s model in the case of cw illumination two different temperature regimes can be reached: a *temperature confinement regime* in which hot-spots of temperature are formed in proximity of the plasmonic nanostructures and a *temperature delocalization regime* where a uniform temperature is obtained throughout the whole nanoarray. In order to discriminate between these two regimes a dimensionless parameter–the 2D confinement parameter *ζ*_2_–was introduced by the authors, defined as ${\zeta }_{2}=\frac{{p}^{2}}{3LR}$, where *p* is the interparticle distance, *L* is the size of the illuminated area and *R* is the nanoparticles’ radius: for *ζ*_2_ << 1 the delocalization regime is reached, while values of *ζ*_2_ > 1 correspond to the regime of temperature confinement. In the present case we get *ζ*_2_ ≅ 10^−3^, which indicates that the full delocalization of the temperature can be reached in the investigated nanoarray. This finding is further supported by the results of our FEM simulations. In Fig. 4b we reported the simulated temperature map of the Ag nanoparticle array, estimated in stationary conditions at the maximum pump power (173 mW). By looking at the color bar on the right, it emerges that at equilibrium the very same temperature is reached throughout the whole NPA and between the silver nanoparticles and the surrounding EuTTA-doped PMMA film. In particular, this result further confirms that EuTTA-doped PMMA thin films represent an accurate thermometric probe to estimate the actual temperature of plasmonic nanostructures. Furthermore, the simulations provided information on the time scales of the thermalization process. In Fig. 4c we plotted the evolution of the simulated temperature, as a function of time, for the three values of the pump laser power: at all the three powers the temperature grows exponentially, with the same time constant *τ*_*th*_ = 0.44 ± 0.01 ms, and thus the sample’s surface reaches the stationary temperature in about 2 ms. During the experiment, the samples were always let thermalize under illumination with the Ar pump laser for about 5 min before taking the PL measurements, that is for a time interval Δ*t* >> *τ*_*th*_. In this way the stationary regime was definitely reached, which further guarantees the accuracy of the temperature estimate obtained by the measurement of the Eu^3+^ luminescence intensity.

As a final remark, we would like to underline that the results of the present method are not restricted to the particular case-study presented. Our samples were designed to have (i) low absorption at the Eu excitation intensity of the N_2_ laser (to avoid unwanted spurious temperature rises), (ii) high absorption at the Ar heating laser. Indeed, if the heating laser matches the samples’ plasmonic resonance, whose absorption cross-section is the highest, the expected temperature variation is the largest. Accordingly, if the working wavelength of the heating laser is out of the main plasmonic resonances, the expected temperature rise will simply scale linearly with the reduced cross-section.

## Conclusion

Rare-earth fluorescence thermometry has been employed to measure the laser-induced plasmon heating of an ordered array of silver nanoparticles synthesized by nanosphere lithography, upon illumination with a cw Ar laser. EuTTA-doped PMMA thin-films deposited by spin-coating on the samples have been used as the thermometric probe, by monitoring the temperature-dependent Eu^3+^ photoluminescent emission in the visible range. Proper thermal treatments of the silver NPA have been performed in order to match the surface plasmon resonance of the nanoarray with the main lines of the pump Ar laser to maximize the photo-induced heating of the samples. In this condition, a maximum temperature increase of ΔT = 19 °C has been determined in the silver nanoarray, upon illumination at the maximum power of the pump Ar laser (173 mW). The experimental findings have been supported by the results of FEM electrodynamic simulations, which provided also information on the temporal dynamics of the heating process: an exponentially-saturated rise in temperature was evidenced at all the three pump powers employed, with a time constant *τ*_*th*_ = 0.44 ± 0.01 ms. In conclusion, rare-earth fluorescence thermometry with EuTTA-doped PMMA thin-films proved to be a facile and accurate method to probe the actual temperature increase due to photo-induced plasmon heating in plasmonic nanosystems. Moreover, the moderate rise in temperature evidenced - even at the highest pump laser power - is interesting particularly considering the use of plasmonic nanoparticles arrays as intensity-interrogated sensors for bio-molecules, which could be denaturated by an excessive temperature increase. Typically lamp sources with a much lower power are used for the measurements with plasmonic biosensors, but the present results indicate that also higher power cw laser can be safely employed.
